# Pre-concentration of microalga *Euglena gracilis* by alkalescent pH treatment and flocculation mechanism of Ca_3_(PO_4_)_2_, Mg_3_(PO_4_)_2_, and derivatives

**DOI:** 10.1186/s13068-020-01734-8

**Published:** 2020-05-29

**Authors:** Mingcan Wu, Jing Li, Huan Qin, Anping Lei, Hui Zhu, Zhangli Hu, Jiangxin Wang

**Affiliations:** 1 Shenzhen Key Laboratory of Marine Bioresource and Eco-environmental Science, Shenzhen Engineering Laboratory for Marine Algal Biotechnology, Guangdong Provincial Key Laboratory for Plant Epigenetics, College of Life Sciences and Oceanography, Shenzhen University, Shenzhen, 518060 China; 2 Key Laboratory of Optoelectronic Devices and Systems of Ministry of Education and Guangdong Province, College of Optoelectronic Engineering, Shenzhen University, Shenzhen, 518060 China; 3 College of Food Engineering and Biotechnology, Hanshan Normal University, Chaozhou, 521041 China

**Keywords:** Microalgae, *Euglena gracilis*, Magnesium phosphate, Calcium phosphate, Harvesting, Flocculation

## Abstract

**Background:**

Microalgae are widely be used in carbon sequestration, food supplements, natural pigments, polyunsaturated fatty acids, biofuel applications, and wastewater treatment. However, the difficulties incurred in algae cell separation and harvesting, and the exorbitant cost required to overcome these challenges, are the primary limitations to large-scale industrial application of microalgae technology.

**Results:**

Herein, we explore the potential of inducing flocculation by adjusting the pH for pre-concentrating *Euglena gracilis*. Our results demonstrate that flocculation can be induced by increasing the medium pH to 8.5; however, most of the algae cells were broken by increasing the pH > 10. Magnesium phosphate, calcium phosphate, and their derivatives precipitation jointly led to flocculation, although calcium phosphate and its derivatives precipitation had a greater effect.

**Conclusions:**

This study demonstrates that pH treatment-induced flocculation is efficient and feasible for the pre-concentration of *E. gracilis* under a pilot-scale culture system. Moreover, it also maintained the microalgae cells’ integrity, chlorophyll production, and increased paramylon production. These findings provide a theoretical basis for reducing the cost of large-scale *E. gracilis* harvesting; as well as provide a reference for harvesting other microalgae.

## Highlights

- 1.It is efficient to pre-concentrate microalga* E. gracilis* by alkalescent pH 8.5 treatment.
- 2.Magnesium phosphate, calcium phosphate and their derivatives precipitation jointly led to flocculation, although calcium phosphate and its derivatives precipitation had a greater effect.
- 3.This flocculation method maintains the *E. gracilis* cell integrity, chlorophyll production , and improves the paramylon production.

## Background

Microalgae exhibit a high biomass production and growth rate and do not compete with crops, as algae can grow on non-arable land [1]. Microalgae biomass may be used in carbon sequestration, food supplements, natural pigments, polyunsaturated fatty acids, biofuel applications, and wastewater treatment [2–6]. Nevertheless, microalgae biomass is generally harvested at < 0.1% solids with cell sizes usually between 3 and 30 μm under large-scale photoautotrophic conditions [7]. To realize the industrial utilization of algal biomass materials, the optimal solids should be > 50%. Therefore, the algal solution under conventional culture needs to be more concentrated and converted into algal mud before being applied in the industry. This separation and concentration process is the main energy consumption expense in microalgae biomass production and generally accounts for 20–60% of the total cost [7]. Thus, the difficulties incurred in algal cell separation and harvesting, and the exorbitant cost required to overcome these challenges, are primary limitations to large-scale industrial application of microalgae technology.

The unicellular flagellated alga, *Euglena gracilis*, which is characterized by the absence of a cell wall, produces a wide variety of bioactive compounds (e.g., paramylon, carotenoids, tocopherol, euglenophycin, and lipids) with tremendous potential for metabolic engineering and commercialization [8]. However, this kind of economic microalgae was pre-concentrated by ultrafiltration membrane filtration [9], which readily results in membrane pollution and subsequently requires a high quantity of water to clean the membrane [10]. At the same time, according to our previous research, when the transmembrane pressure was slightly higher (0.2 MPa), the *E. gracilis* cells easily ruptured, leading to a low recovery efficiency and poor cost improvement. Therefore, a more economical and efficient method of pre-concentrating *E. gracilis* cells is urgently needed.

Generally, microalgae cells are concentrated by centrifugation [11]. However, due to the high energy consumption requirement, centrifugation cannot be directly used in large-scale engineering. Therefore, algae cells need to be pre-concentrated. Algae cells can be pre-concentrated through various methods, including the electrolytic method [12], foam fractionation [13], gravity sedimentation [14], ultrafiltration membrane filtration [15], and flocculation [16]. In particular, flocculation is a promising approach to reducing the cost of microalgae harvesting, as it is an effective, yet simple technique. Flocculation enables the rapid separation of microalgae from the medium using simple gravity-based sedimentation [17]. Although the algae cells do not meet industrial application requirements directly after flocculation–sedimentation, the inclusion of this technique can significantly reduce the energy consumption and cost of subsequent concentration processing. Therefore, flocculation is regarded as the best way to achieve large-scale separation and pre-concentration of microalgae [10]. Moreover, adjusting the medium’s pH to > 8 leads to algae cell flocculation without the need for additional flocculant. However, the mechanism that drives this process is not yet clearly understood. A small group of researchers supports the notion that when the medium is weak alkaline (pH 8–10), a large number of positively charged calcium phosphate precipitates are formed, which can neutralize the negatively charged algae cells [7, 18]; however, Branyikova et al. [19] found that flocculation of negatively charged *Chlorella vulgaris* was induced not only by positively charged but also by negatively charged calcium phosphate precipitates at an early phase of nucleation. The driving force for interactions between oppositely charged cells and precipitate particles was electrostatic attraction, while the attraction between equally charged entities may have resulted from a negative total balance of apolar (Lifsitz–van der Waals) and polar (acid–base) interactions. By contrast, most researchers support the view that when the medium pH is > 10.5, it can produce positive magnesium hydroxide precipitation, neutralize the algae cells with a negative charge, and then form flocculation [1, 16, 20–22]. In this case, magnesium hydroxide, as opposed to calcium phosphate, is the key precipitate that induces algae cell flocculation. Moreover, Formosa-Dague et al. [23] suggested that the magnesium-hydroxide flocculation mechanism of *Phaeodactylum tricornutum* is a two-partner system, both the changes are undertaken by the cell wall and the interactions with hydroxide particles are needed to successfully flocculation. Due to this unresolved controversy, the flocculation mechanism of no-cell wall algae, *E. gracilis,* is required elucidation.

Nevertheless, the activity and subsequent utilization of algae cells are often adversely affected by a high pH. For example, the high-yield lipid algae species *Skeletoma costatum* reached 80% flocculation efficiency at pH 10 after 2 h, but a considerable part of the cells in the recovered algae body disintegrated and the intracellular components leaked out, which drastically affected the subsequent eicosapentaenoic acid (EPA) extraction process [24]. When the pH of *Spirulina platensis* suspensions was > 13, the cells flocculated quickly and thoroughly, but the cells’ pigment turned yellow, indicating that the “pH” seriously damaged the cells [25]. Also, high pH value is likely to exceed the effluent discharge standard; thus the pH must be adjusted to within the allowable range. Therefore, the optimal harvesting technique should be independent of the cultured species, consume little energy and chemicals, and harmless to the valuable products obtained during the extraction process [26].

In this study, we attempted to (1) determine the optimal flocculation pH value by adjusting the suspension pH with sodium hydroxide solution (NaOH) and hydrochloric acid (HCl) to induce flocculation of *E. gracilis* cells; (2) investigate potential flocculation mechanisms; and (3) verify and evaluate the method of pre-concentration of *E. gracilis* and cells’ biochemical components under a pilot-scale culture, respectively.

## Results and discussion

### The growth curve of *E. gracilis* and pH changes in culture conditions

**Fig. 1:**
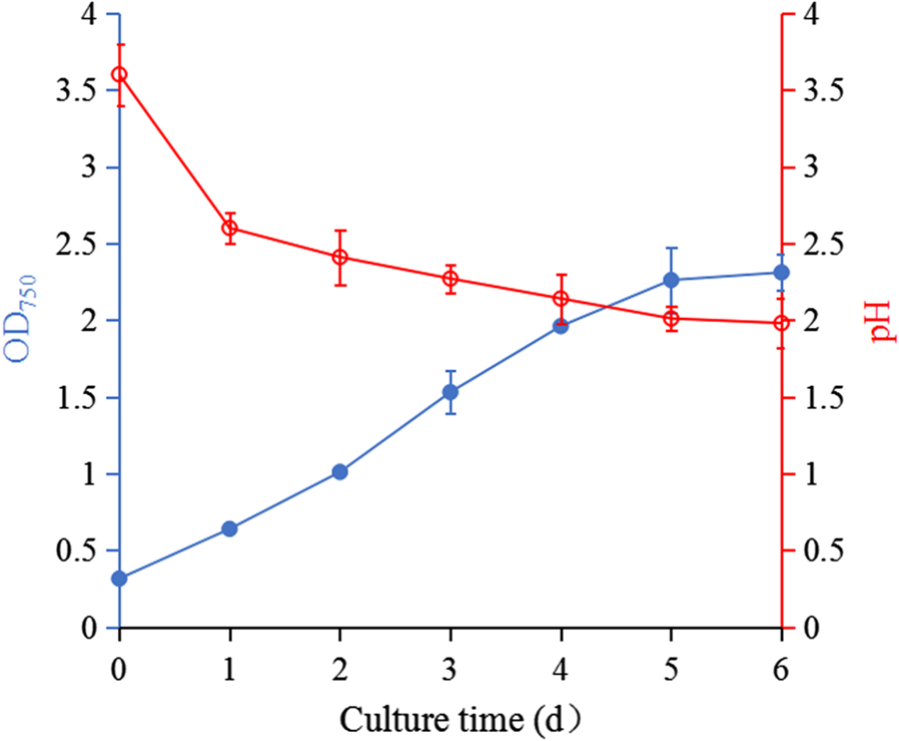
Growth curve of *E. gracilis* and pH changes under photoautotrophic conditions. OD_750_ represents biomass, and pH the varying pH value of the PEM medium. The values represent mean ± S.D. *n* = 3

Under photoautotrophic conditions, generally, the pH gradually increases as algae cells grow; thus, pH levels are > 7 throughout the culture process. As such, CO_2_ is often used to control the pH within an appropriate range to prevent a decrease in photosynthetic efficiency [27, 28]. However, the varying pH culture conditions of *E. gracilis* differ from many microalgae. For example, the strain’s maximum biomass is 2.3 by OD_750_ under photoautotrophic conditions, yet the medium’s pH gradually decreased from the original 3.6, reaching 1.9 in the platform period by day 6 (Fig. 1). This result suggests that *E. gracilis* is always growing in an acidic environment.

### pH-induced flocculation

Microalgae flocculation in the absence of flocculant is generally referred to as “auto-flocculation”. Essentially, the algae cells’ high photosynthetic efficiency facilitates large quantities of CO_2_ absorption, which increases the algae suspensions’ pH. When the medium’s pH is raised to a certain threshold without CO_2_, positively charged precipitates (mainly calcium phosphate) are formed from specific metal ions in the algae suspensions, which can neutralize the algae cells’ negatively charged surface, and enable flocculation [18]. In contrast to “auto-flocculation”, flocculation can also be artificially induced by adding alkaline substances to improve the algae suspensions’ pH. Based on the above results, the medium with *E. gracilis* cells is acidic. According to Ozkan [29] and Liu et al. [30], the algae cells’ surface is generally positively charged when algae cells are in acidic conditions. Therefore, *E. gracilis* harvesting is unable to be performed with auto-flocculation. As such, the medium’s pH must be artificially increased, by adding alkaline substances, to convert the algae cell surfaces from positive to negative charges.

**Fig. 2:**
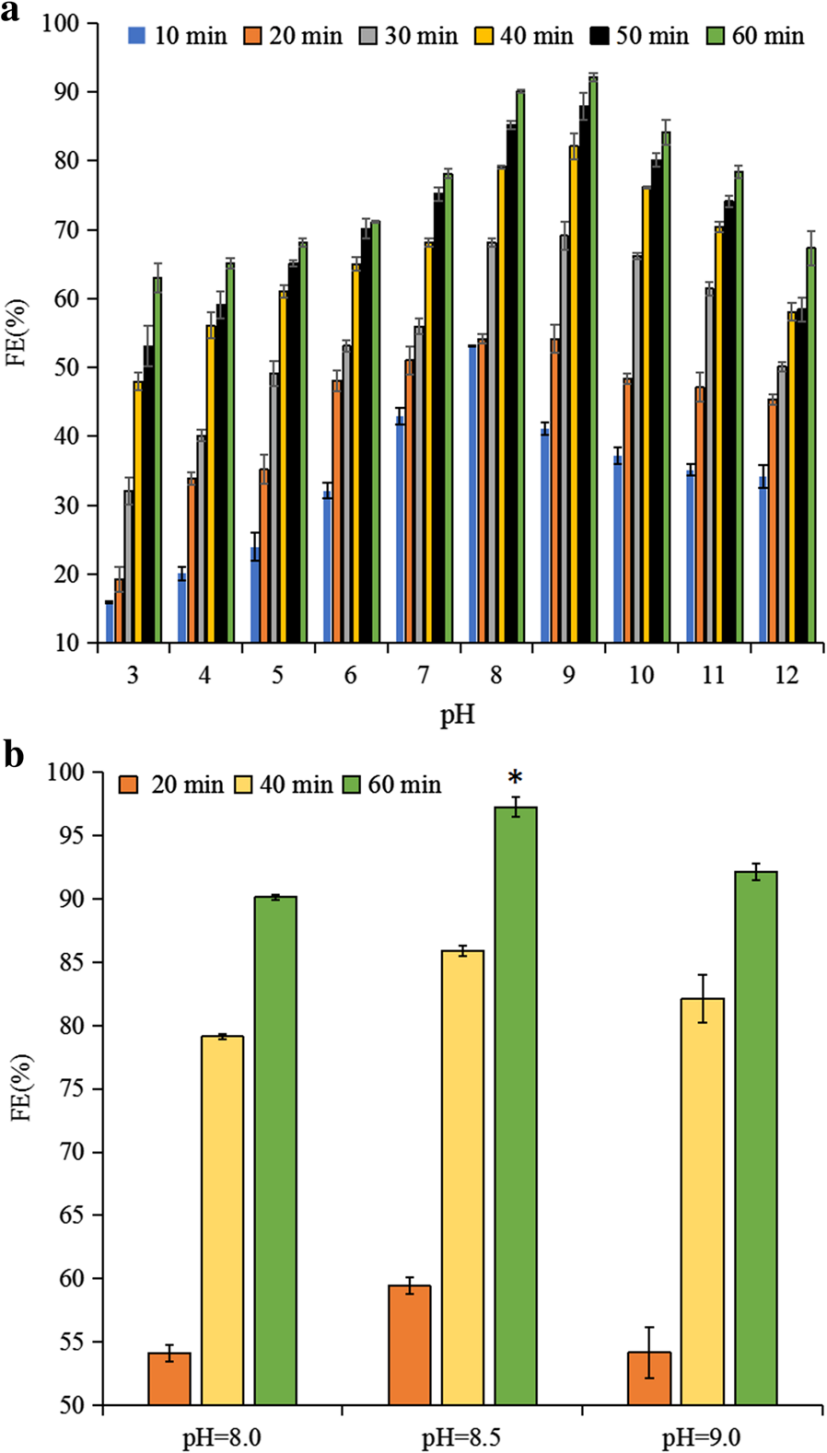
Flocculation efficiency of *E. gracilis* under varying pH treatments. **a** The FE was determined over a range of pHs. **b** The FE was used to determine the critical point of pH value. *FE* flocculation efficiency; * represents *p *< 0.05. The values represent mean ± S.D. *n* = 3

The pH of the *E. gracilis* cells’ culture medium was adjusted by adding NaOH and HCl solution. An extended time analysis showed that algae cells achieved a greater flocculation efficiency (FE) (> 80%) at pH 8, 9, and 10 (Fig. 2a, Additional file 1: Fig. S1). What’s more, based on the algae cells’ FE at pH 8, 8.5, and 9, the critical pH value (CPV) was determined as 8.5 (*p* < 0.05) (Fig. 2b). These results are similar to those specific results given by Beuckels et al. [7] about harvesting the freshwater microalgae *C. vulgaris*; although, it has been suggested that the pH adjustment treatment is more efficient for harvesting *E. gracilis* cells.

**Fig. 3:**
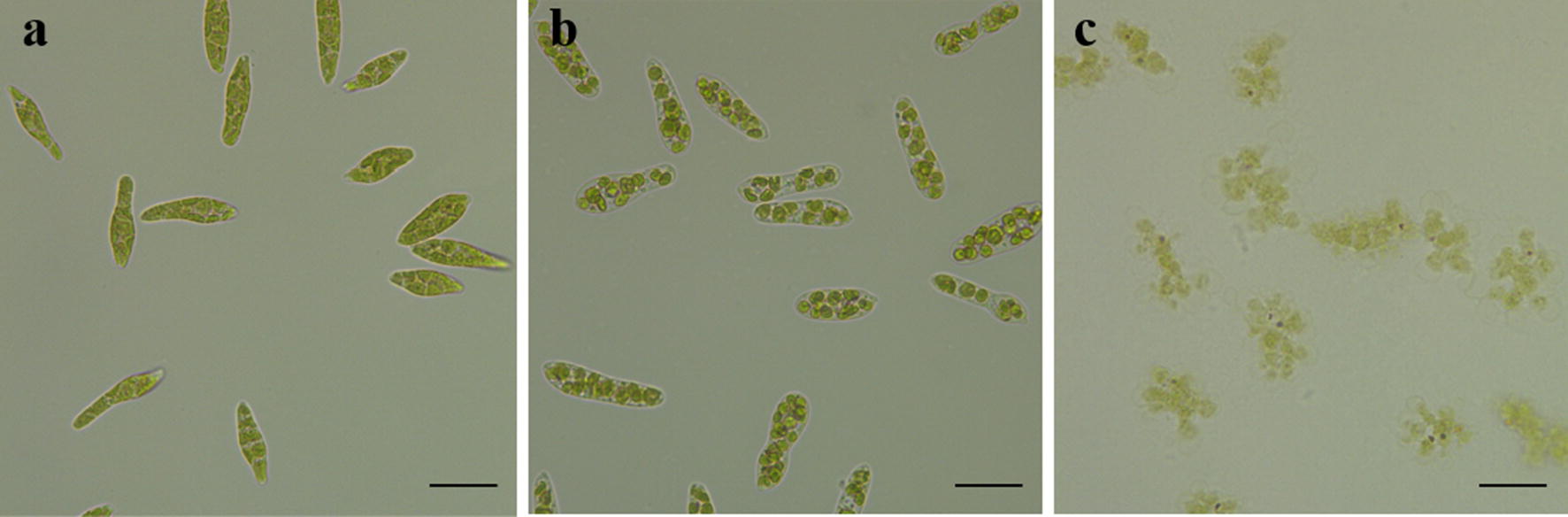
The morphological changes of *E. gracilis* cells under different pH values. Morphology of algae cells at pH: **a** 3.5, **b** 8.5, and **c** > 10. The scale bar represents 20 μm

However, when the pH value was > 8.5, the FE gradually decreased (Fig. 2a). Unexpectedly, microscopic observation of the *E. gracilis* cells’ morphology showed significant algal cell morphological changes, based on the medium’s pH level. For example, at pH 3.5, the cells were slender, and the chloroplasts were flat (Fig. 3a); whereas, at pH 8.5, the chloroplasts were spherical (Fig. 3b). At pH > 10, the algal cells were completely ruptured and the cells’ components were suspended in the liquid (Fig. 3c, Additional file 1: Fig. S1). This phenomenon rarely occurs in other green algae, such as *C. vulgaris* and *Scenedesmus obliquus* [7, 16, 20]. A potential explanation for the cell rupture may be related to the fact that the *E. gracilis* cells have no cell wall. Due to the absence of cell wall support, when the osmotic pressure became too intense, the cell ruptured, obviously indicating that *E. gracilis* cells are unable to flocculate at higher pHs.

**Fig. 4:**
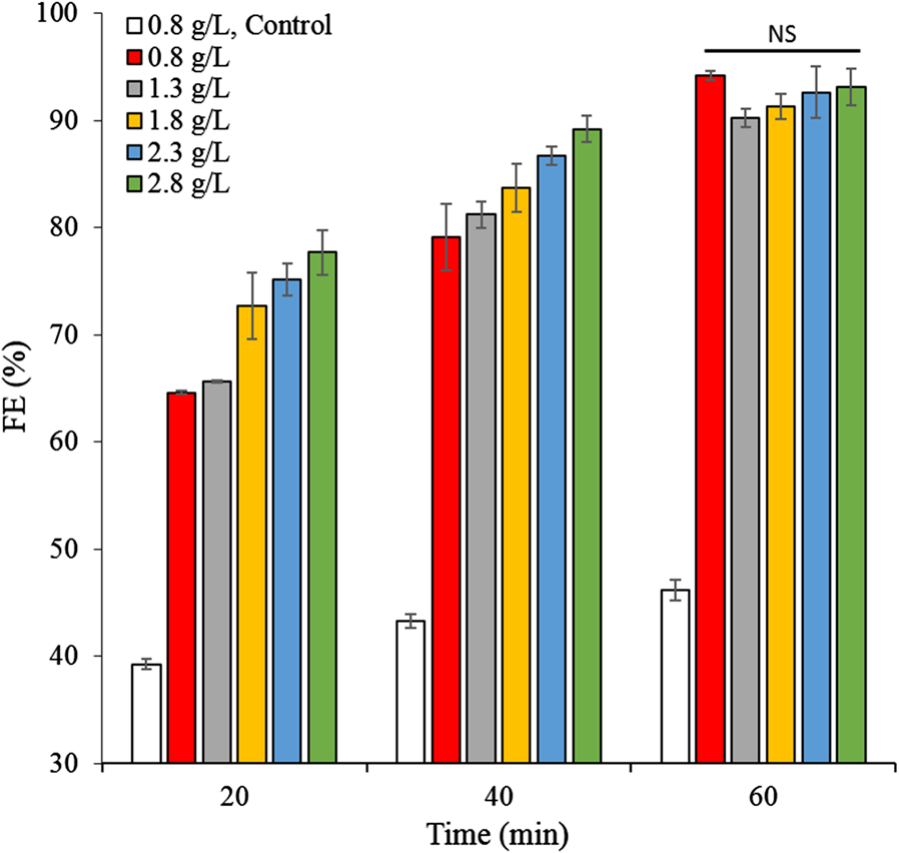
The FE of samples with varying *E. gracilis* cell biomass. *FE* flocculation efficiency, *NS* represents *p *> 0.05. The values represent mean ± S.D. *n* = 3

To investigate the effect of the CPV on *E. gracilis* cell biomass, the cells’ FE was measured at 0.8, 1.3, 1.8, 2.3, and 2.8 g/L. The results showed that the FEs of all the different cell densities reached > 90% after 1 h, with no significant difference between them (*p* > 0.05) (Fig. 4). As such, the CPV has no distinctive impact on cell growth of varying biomass densities; possibly because key ions in the culture medium are always redundant, although some of them are absorbed by algae cells during the culturing process.

### Mechanism of the flocculation

**Fig. 5:**
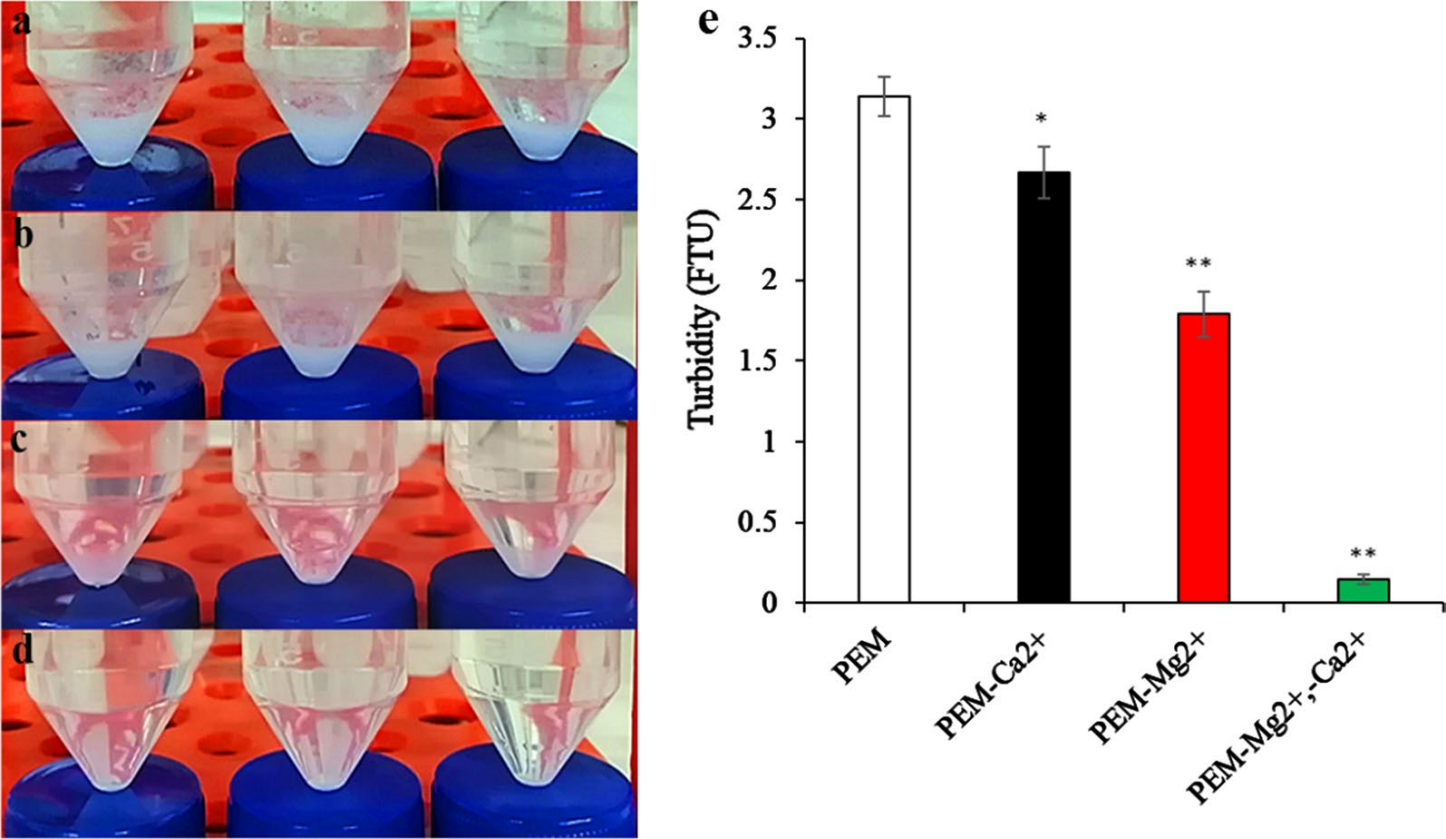
The visual quantification of precipitates and turbidity were determined in the PEM medium by pH 8.5 treatment. **a** Control group under fresh PEM medium at pH 8.5; PEM without **b** Ca^2+^, **c** Mg^2+^, and **d** both Ca^2+^ and Mg^2+^; **e** turbidity was determined under different conditions; PEM represents control group, fresh PEM medium; PEM-Mg^2+^ represents PEM without MgSO_4_·7H_2_O; PEM-Ca^2+^ represents PEM without CaCl_2_·2H_2_O; PEM-Mg^2+^,-Ca^2+^ represents PEM without MgSO_4_·7H_2_O, CaCl_2_·2H_2_O; * and ** represent *p *< 0.05, *p *< 0.01, respectively. The values represent mean ± S.D. *n* = 3

When the algal suspension’s pH was adjusted to 7, precipitate generation gradually began. Thus, at pH 10, after 1 h, there was a sizeable amount of deposited precipitate at the bottom of the centrifuge tube containing the suspension (Additional file 1: Fig. S1). Simultaneously, we found that Mg^2+^ and Ca^2+^ exhibited the highest and second-highest metal ion concentration in the PEM medium (Additional file 1: Table S1). Therefore, this study focused on the role of these two metal ions. To reduce interference in the precipitate analysis from the algal cells’ pigment, the experiments were performed in a fresh medium as opposed to previously prepared algal suspensions. After the fresh culture medium’s pH had been adjusted to 8.5 for 1 h, a large number of white precipitates were deposited at the bottom of the centrifuge tube (Fig. 5a). Interestingly, while precipitates were produced when Ca^2+^ was absent from the culture medium (Fig. 5b), only a small number of precipitates were deposited when Mg^2+^ was absent (Fig. 5c). When both Ca^2+^ and Mg^2+^ were absent, no precipitate formed in the culture medium (Fig. 5d). Meanwhile, the turbidity of the solutions from the central of centrifuge tubes was 3.2,2.6,1.7, and 0.2 FUT under PEM, PEM-Mg^2+^, PEM-Ca^2+^, and PEM-Mg^2+^, -Ca^2+^ conditions, respectively (Fig. 5e). These results demonstrated that precipitate formation was primarily controlled by the presence or absence of Ca^2+^ and Mg^2+^.

**Fig. 6:**
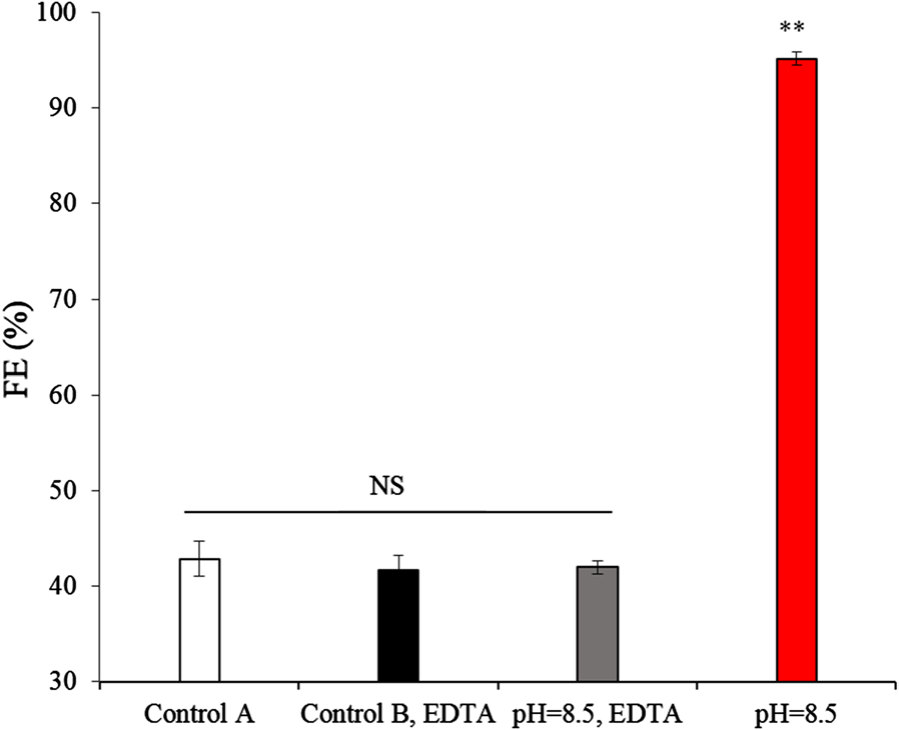
FE of *E. gracilis* was determined at pH 8.5 with and without EDTA. FE, flocculation efficiency; EDTA, ethylene diamine tetraacetic acid. Control A represents a control group at the CPV without EDTA; Control B represents a control group with added EDTA; NS, ** represent *p* > 0.05, *p *< 0.01, respectively. The values represent mean ± S.D. *n* = 3

To unequivocally demonstrate the role these bivalent cations play in the flocculation process, all-metal ions in the PEM medium were chelated with a saturated concentration of EDTA. The results showed that the pH 8.5 algae solution supplemented with EDTA depicted no significant difference in the FE compared to the control group (*p *> 0.05). However, the same algae solution with no EDTA depicted an FE increase ranging from 42.2 to 93.3% (*p* < 0.01) (Fig. 6). Thus, it confirmed that the metal ions such as Ca^2+^ and/or Mg^2+^ were the main cause of flocculation; the results were similar to the previous study except for those algae solutions at > pH 10.5 by Vandamme et al. [16].

**Fig. 7:**
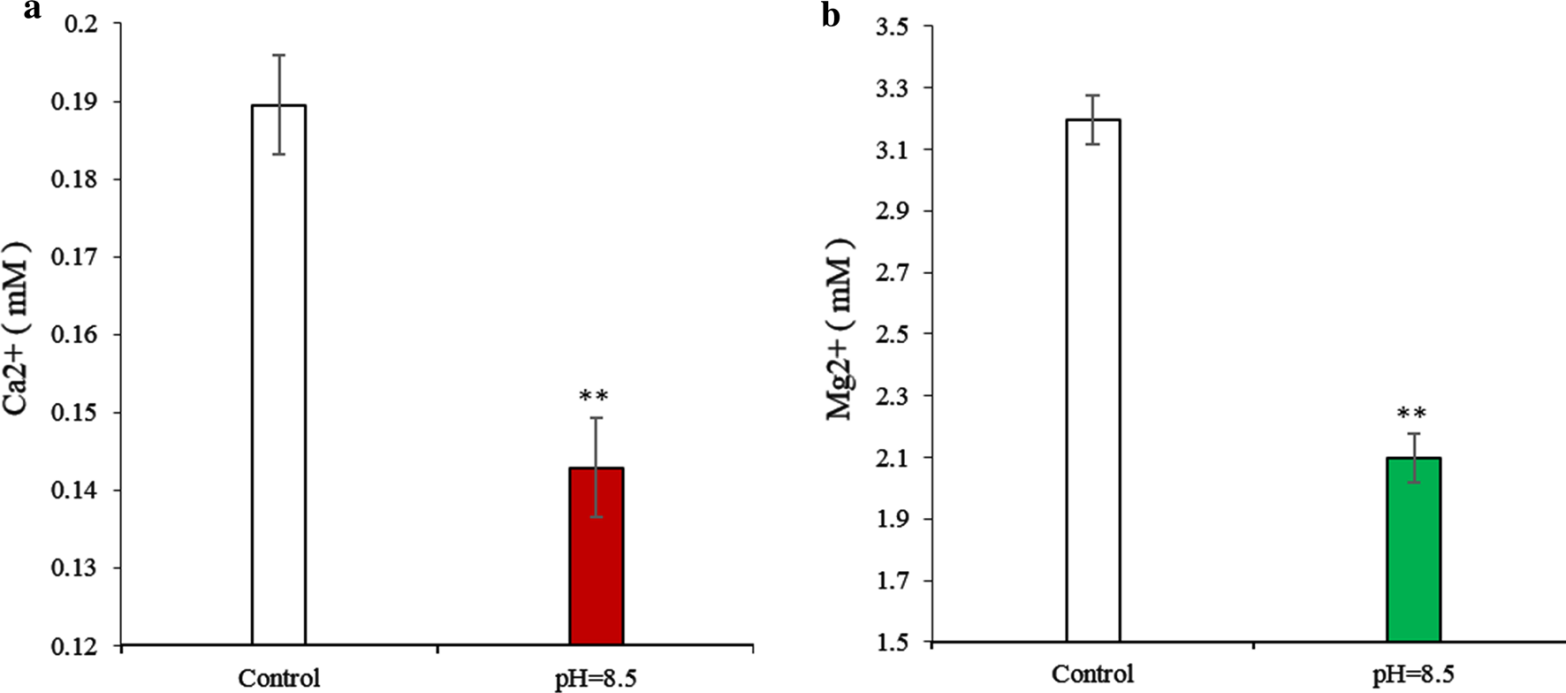
The concentration change of dissolved **a** Ca^2+^ and **b** Mg^2+^ in the PEM medium adjusted to pH 8.5. Control represents the medium without pH treatment. NS, ** represent *p* > 0.05, *p *< 0.01, respectively. The values represent mean ± S.D. *n* = 3

After the algae cells underwent flocculation–sedimentation, the Ca^2+^ and Mg^2+^ concentration in the culture medium decreased from 0.19 mM to 0.14 mM, and 3.2 mM to 2.1 mM, respectively, thereby depicting a significant (*p* < 0.01) decrease of 26.3% and 34.4%, respectively (Fig. 7). Meanwhile, we predicted and analyzed the compounds synthesized by these two metal ions at pH 8.5 through Visual MINTEQ software, and found that there were as many as 7 compounds, that were included of 3 variants of Ca_3_(PO_4_)_2_ (β-Ca_3_(PO_4_)_2_, am_1_-Ca_3_(PO_4_)_2_, am_2_-Ca_3_(PO_4_)_2_) and 4 derivatives (Ca_10_(PO_4_)_6_(OH)_2_, Ca_4_H(PO_4_)_3_·3H_2_O, CaHPO_4_, CaHPO_4_·2H_2_O) related to calcium ion synthesis with SI (Saturation index) > 1, and only both Mg_3_(PO_4_)_2_ and MgHPO_4_·3H_2_O, that were included of related to magnesium ion synthesis (Table 1). It showed that these two metal ions may be more inclined to form a precipitate with phosphate at such a pH value. Thus, these two metal ions may simultaneously play a role in algae cell flocculation. This result conflicts with that of Sukenik et al. [18, 31], who reported that when the pH was adjusted to 8.9, the Ca^2+^ concentration decreased, while that of Mg^2+^ remained unchanged. In this study, we found that magnesium ion can also form precipitates at the CPV under an alkalescent pH.

**Table 1:** The saturation indices (SI) of the different compounds in the PEM medium by pH 8.5, 25 °C treatment were predicted with Visual MINTEQ software

Compounds	lgIAP	lgKsp	SI^a^
Ca_10_(PO_4_)_6_(OH)_2_	− 20.90	− 44.33	23.43^b^
Ca_4_H(PO_4_)_3_·3H_2_O	− 34.33	− 47.95	13.63^b^
β-Ca_3_(PO_4_)_2_	− 18.41	− 28.92	10.51^b^
am_2_-Ca_3_(PO_4_)_2_	− 18.41	− 28.25	9.84^b^
Mg_3_(PO_4_)_2_	− 14.08	− 23.28	9.20^b^
am_1_-Ca_3_(PO_4_)_2_	− 18.41	− 25.50	7.09^b^
MgHPO_4_·3H_2_O	− 14.47	− 18.18	3.70^b^
CaHPO_4_	− 15.92	− 19.28	3.36^b^
CaHPO_4_·2H_2_O	− 15.92	− 19.00	3.08^b^
Mg(OH)_2_	14.87	18.79	− 3.93^c^
MgO	14.87	21.58	− 6.72^c^
Ca(OH)_2_	13.42	22.70	− 9.28^c^
CaO	13.42	32.70	− 19.28^c^

^*a*^*SI* saturation index = lgIAP − lgKsp; ^b^SI > 1, oversaturation; ^c^SI < 1, undersaturation; *IAP* ion activity product, *K*_*SP*_ solubility product constant

**Fig. 8:**
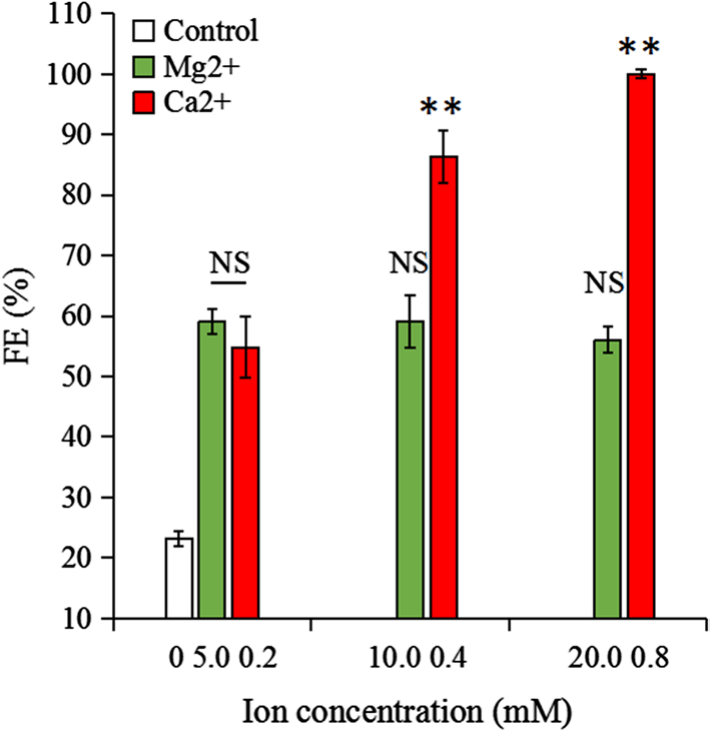
FE of *E. gracilis* was determined at pH 8.5 with different concentrations of Ca^2+^ and Mg^2+^. FE, flocculation efficiency. Control represents fresh PEM medium without Ca^2+^ and Mg^2+^. NS, ** represent *p* > 0.05, *p *< 0.01, respectively. The values represent mean ± S.D. *n* = 3

**Fig. 9:**
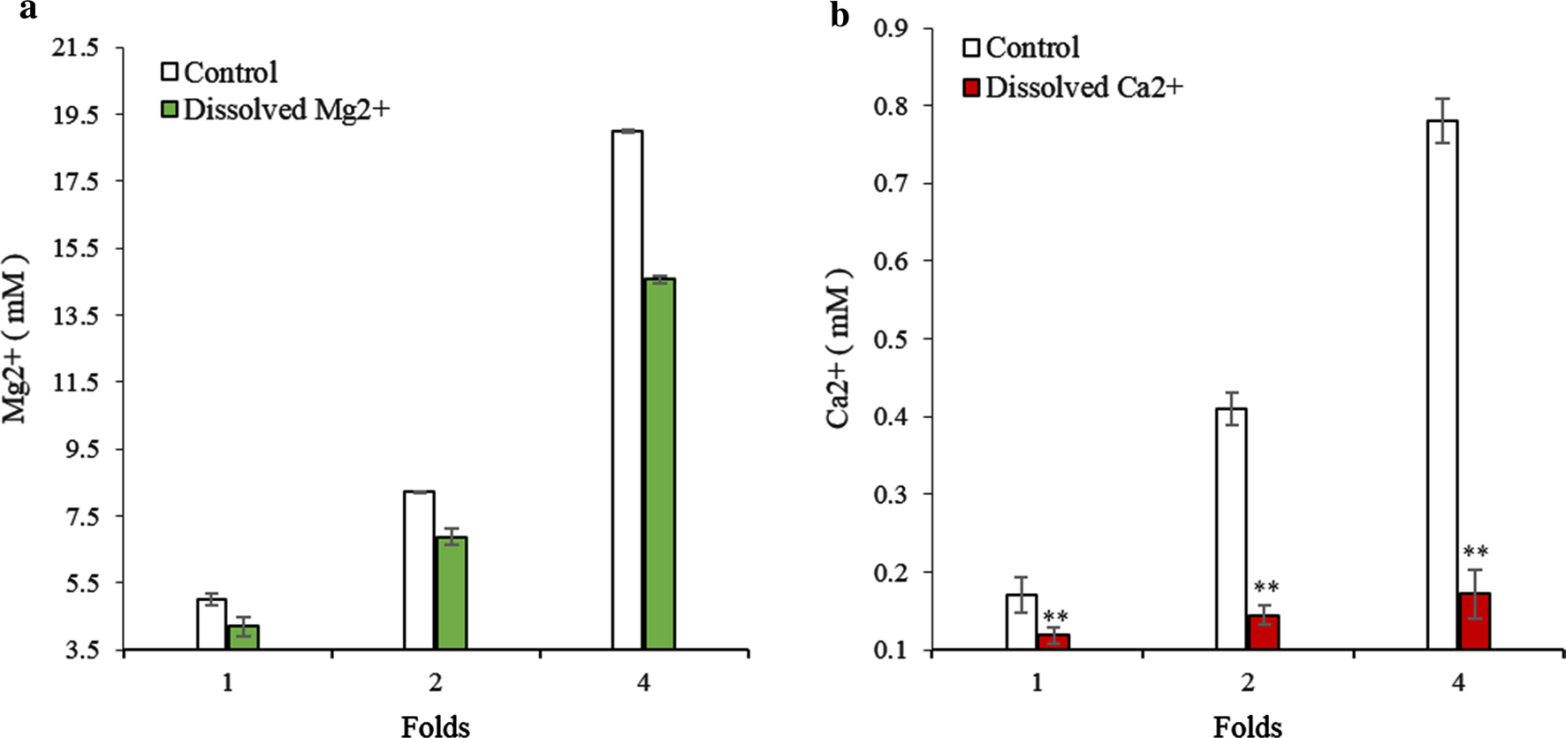
The concentrations of dissolved Ca^2+^ and Mg^2+^ in the PEM medium adjusted to pH 8.5 after adding multiple fold concentrations of Ca^2+^ and Mg^2+^. Dissolved **a** Mg^2+^ and **b** Ca^2+^ concentrations were quantified, respectively. Control was without pH adjustment. ** represent *p* < 0.01. The values represent mean ± S.D. *n* = 3

To more specifically determine which of the two divalent metal cations plays a more significant role, different concentrations of Ca^2+^ and Mg^2+^ were added to the algal cell suspensions. The results showed that when the Mg^2+^ and Ca^2+^ concentration was doubled (5 mM Mg^2+^, 0.2 mM Ca^2+)^, the FE was 59.1% and 54.8%, respectively, meaning that neither ion by itself could induce algae cell flocculation. Interestingly, regardless of how much Mg^2+^ was added, the FE remained at 56–59% (*p* > 0.05); however, the FE gradually increased in response to an increasing Ca^2+^ concentration. In fact, the FE reached 100% (*p* < 0.01) after the Ca^2+^ concentration was increased fourfold (20 mM Mg^2+^, 0.8 mM Ca^2+^) (Fig. 8). Besides, the dissolved Ca^2+^ and Mg^2+^ in the fresh medium were analyzed by ICP-OES. Results showed that the dissolved Mg^2+^ concentration gradually increased with the addition of Mg^2+^ (Fig. 9a). In contrast, the concentration of dissolved Ca^2+^ remained between 0.1 and 0.2 mM (*p* < 0.05) (Fig. 9b) regardless of how much calcium ion was added. The combined results depicted in Figs. 8 and 9 confirm that Ca^2+^ and Mg^2+^ play comparable key roles in the flocculation of algae cells at the CPV.

**Fig. 10:**
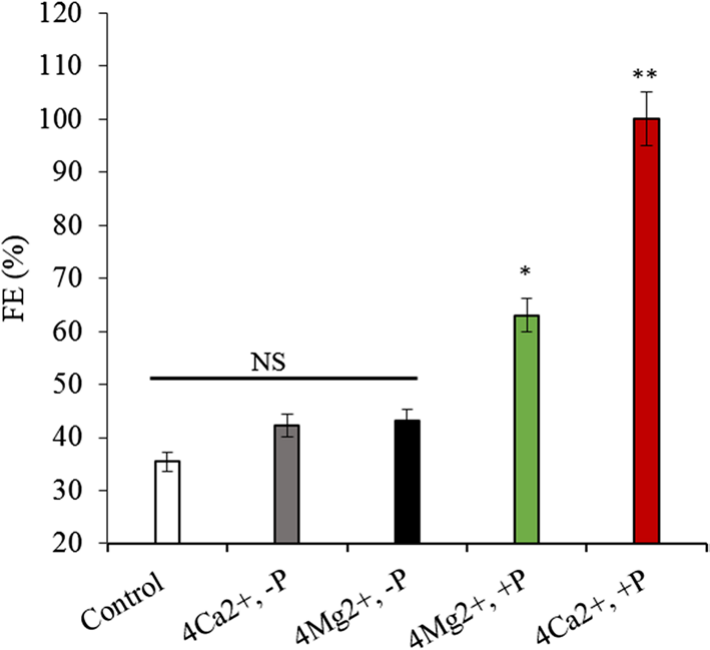
Effect of Ca^2+^, Mg^2+^, and PO_4_^3−^ on FE in *E. gracilis* suspensions. 4Ca^2+^ and 4 Mg^2+^ represent fourfold concentration of Ca^2+^ (0.8 mM) and Mg^2+^ (20.0 mM), respectively; −*P* and +*P* represent without and with PO_4_^3−^ (4.4 mM), respectively. Control represents medium without Ca^2+^, Mg^2+^, and PO_4_^3−^; NS, *, and ** represent *p* > 0.05, *p *< 0.05, and *p *< 0.01, respectively. The values represent mean ± S.D. *n* = 3

Next, we set out to determine what anions combined with the Ca^2+^ and Mg^2+^ to form sediment, and ultimately induce algae cell flocculation. Based on the published literature, calcium phosphate precipitates easily form [7, 18] when there is a high PO_4_^3−^ concentration and the medium’s pH is weak alkaline. In our study, we found that when a fourfold (0.8 mM) concentration of Ca^2+^ was added to the medium without onefold (4.4 mM) PO_4_^3−^, the FE was only 42.24%, and there was no significant difference (*p* > 0.05) compared with the control group (35.35%). Contrarily, when PO_4_^3−^ was present, the FE reached 100%. Notably, we also found that when a fourfold (20.0 mM) concentration of Mg^2+^ was added to the medium without PO_4_^3−^, the FE was 43.12%; and when PO_4_^3−^ was added, the FE only increased to 62.98% far lower than the effect observed with Ca^2+^ (Fig. 10). Through Visual MINTEQ software prediction and analysis, the logarithmic Solubility Product Constants (lg Ksp) of the solubility products of the three calcium phosphate derivatives β-Ca_3_(PO_4_)_2_, am_1_-Ca_3_(PO_4_)_2_, and am_2_-Ca_3_(PO_4_)_2_ were − 28.92, − 25.2, and − 28.25, respectively, which are lower than − 23.28 of magnesium phosphate. The lg Ksp of the derivatives are − 44.33, − 47.95, − 19.28, and − 19.00, which are also lower than − 18.18 of the magnesium phosphate derivative MgHPO_4_·3H_2_O, and the SI of all compounds were > 1 (Table 1), meaning calcium phosphate and their derivatives were easier to form than magnesium phosphate and their derivatives. These results verify that the flocculation was caused by calcium phosphate, magnesium phosphate and both their derivatives’ precipitate formation, in which the calcium phosphate and derivatives’ effect plays a larger role.

Although the extended DLVO (XDLVO) theory is traditionally used to describe colloidal stability in colloidal chemistry, it also suitably describes the aggregation process of algae cells in microalgae suspension [29, 32, 33]. According to this theory, the interaction forces within algae cell suspensions include the Lifshitz–van der Waals interaction (LDWI), electrostatic interaction (EI), and lewis acid–base interaction (LABI). In general, LDWI is an attraction force. In pH > 7 microalgae culture systems, negative charges develop on the algae cells’ surface, so the EI behaves as a repulsion force [29]. Most algae cells are hydrophilic and thus have a tendency to disperse in aqueous solution. As such, the LABI behaves as a repulsion force [29, 33]. In essence, algae suspensions are stabilized through a combination of all three forces. Therefore, the basic principle of microalgae flocculation is to reduce or eliminate the repulsive EI and LABI forces, so that algae cells can aggregate and form flocculation.

Therefore, to induce algae cell flocculation by adjusting the medium pH, the main ions’ types in the medium and the resulting precipitates must be identified. Auto-flocculation induction only occurs if there is enough PO_4_^3−^ and Ca^2+^ in the medium from the start. When the algae solution pH is weakly alkaline (8–10), positively charged sediment can be formed to neutralize the negative charge on the algae cells’ surface, which enables algae cell flocculation [18]. However, most studies report that microalgae culture mediums generally contain an overabundance of PO_4_^3^ and Mg^2+^, as opposed to PO_4_^3−^ and Ca^2+^ [1, 16, 20–22], which prevents it from flocculating under weak alkalinity. Large quantities of positively charged magnesium hydroxide only form when the algae solution’s pH value is very high (> 10.5). Once precipitated, they can neutralize the negative charge on the algae cells’ surface; and then physical methods, such as net catching and sweeping, can be used to accelerate the gravity precipitation and harvest the algae cells.

As previously discussed, the algae cell flocculation mechanism differs depending on the ion and pH value present in the suspension. *E. gracilis* cells’ PEM medium contained a high concentration of PO_4_^3−^ and Mg^2+^, and a small concentration of Ca^2+^. The CPV in the algae suspension was determined to be 8.5, which is considered weak alkaline; thus flocculation and sedimentation can occur.

In this work, we found that Mg^2+^ formed magnesium phosphate precipitates and induced a flocculation effect at a weakly alkali pH of 8.5, without raising the pH high enough to form magnesium hydroxide precipitate. However, magnesium phosphate’s flocculation effect is limited at such CPV. Regardless of the magnesium ion concentration, the flocculation efficiency essentially remains the same; perhaps because the magnesium ion can promote movement of *E. gracilis* cells [34] and subsequently reduce the FE.

Although most of the precipitates were magnesium phosphate and its derivatives, except for a small amount of calcium phosphate and its derivatives, the occurrence of algae cell flocculation is influenced by both metal ions’ precipitate, as opposed to only calcium phosphate precipitates as previously reported [7, 18]. Thus, the calcium phosphate sediment still plays a key role, as it can neutralize the negative charge, and maybe net catch and sweep cells by a lot of derivatives, and also inhibit *E. gracilis* cell movement [34]. Therefore, this mechanism provides a theoretical basis for harvesting other algae, and for optimization of microalgae culture mediums conducive to flocculation.

### Pilot-scale culture performance and chemical compounds

**Fig. 11:**
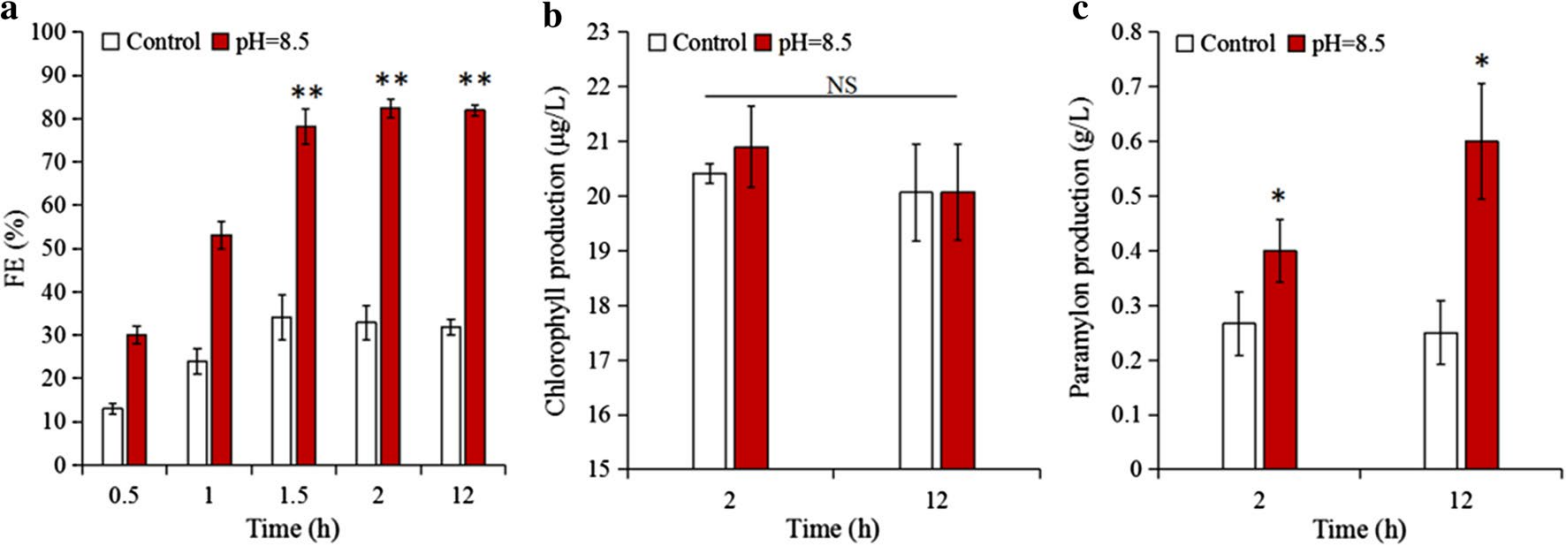
Preliminary harvesting of *E. gracilis* cells by pH treatment and the effects on chemical compounds under pilot-scale culture. **a** FE of *E. gracilis* cells under pilot-scale culture; **b** chlorophyll and **c** paramylon production; *, ** represent *p* < 0.05, *p *< 0.01, respectively. The values represent mean ± S.D. *n* = 3

To verify the feasibility of harvesting *Euglena gracilis* cells by adjusting the CPV in a pilot-scale culture, we used an *E. gracilis* pre-concentration from the 60 L column photobioreactors. The FE reached 82.4%, 2.5-fold that of the control group (32.9%) (*p *< 0.01) by 2 h (Fig. 11a), clearly demonstrating the positive effect of pre-concentrating *E. gracilis* cells. Thus, it has been suggested that this is a feasible method for harvesting *E. gracilis* cells in a pilot-scale culture. Flocculation of other microalgae requires a high pH (> 10) to achieve a good FE [1, 16, 20–22]. However, in this study, only a small amount of sodium hydroxide was necessary to adjust the pH to 8.5, and the resulting flocculation achieved good results. The pre-concentrated algae suspensions were successfully concentrated by centrifugation after adding a small amount of acid to restore the acidic medium. Thus, pre-concentrating greatly reduces the energy and monetary cost. Yet, the mechanism studied in the laboratory-scale culture cannot always reach the desired FE in the pilot-scale cultures. For example, in the laboratory-scale level experiments, the FE reached > 90% within a short time; whereas, the FE was only 82.4% in the pilot-scale culture that was conducted for 2 h. There are numerous additional influencing factors, such as the dissolved organic matter (DOM) secreted by algae cells, which can significantly inhibit flocculation. These DOM may take the form of humic acid produced by cell rupture, an organic acid produced by algae metabolism, or organic acid secreted by bacteria, etc. [16, 35–38]. Naturally, different algae species cultured under varied conditions may have different primary inhibitors to flocculation. Thus, it is important to determine which abiotic factors can reduce the FE of *E. gracilis* cells, as it will provide a theoretical basis for harvesting *E. gracilis* cells on a larger scale in the future.

Generally, the algae cells’ biochemical substances (such as pigment) were destroyed by a high pH [25], but whether those biochemical substances are also destroyed by a weak alkaline pH remains unknown. The fact that there was no significant change in chlorophyll production when compared with the control group after 2 and 12 h of flocculation (*p* > 0.05) (Fig. 11b) indicates that the CPV had no effect on chlorophyll production, yet it produced a good harvesting effect compared with results in the literature [25]. What’s more, the paramylon production was significantly higher than that of the control group. The highest production was 0.6 g/L after 12 h (*p* < 0.05), which is 2.4-fold that of the control group (Fig. 11c). The results are similar to those specified given by Masahiro et al. [39]. These results verify that paramylon synthesis is induced by pH treatment. In summary, this method not only enables feasible harvesting of *E. gracilis* cells, but also scarcely affects the chlorophyll production, and thereby increases the paramylon production from pH treatments. These benefits will provide solid theoretical guidance for the production of active substances from industrial-size cultures of *E. gracilis* and other microalgae.

## Conclusion

Our study demonstrates that pre-concentrating microalga *E. gracilis* via alkalescent pH 8.5 treatment is both productive and cost-efficient. Essentially, *E. gracilis* cells’ flocculation is influenced by a combination of calcium phosphate, magnesium phosphate, and their derivatives. Calcium phosphate and its derivatives’ precipitate play a key role in the flocculation process, although very little concentration is needed, as it has the greatest impact on flocculation efficiency. This flocculation method has the potential to optimize flocculation conditions in the future, as it was validated in a pilot-scale culture system. Finally, this method maintains the algae cell integrity, chlorophyll production, and improves paramylon production. These findings will both reduce the cost of industrially harvesting *E. gracilis* cells, and provide a theoretical basis for harvesting other microalgae.

## Materials and methods

### Strain and growth conditions

*Euglena gracilis* CCAP 1224/5Z was purchased from the Culture Collection of Algae and Protozoa (CCAP) and maintained in our lab at Shenzhen University [40]. This strain was cultured in a modified photoautotrophic euglena medium (PEM), according to Cramer and Myers [41]. The PEM medium contained 1.8 g/L NH_4_Cl, 0.6 g/L KH_2_PO_4_, 1.2 g/L MgSO_4_·7H_2_O, 0.02 g/L CaCl_2_·2H_2_O, 0.55 μg/L Na_2_EDTA·2H_2_O, 2 μg/L Fe_2_(SO_4_)_3_, 0.05 μg/L CuSO_4_·5H_2_O, 0.4 μg/L ZnSO_4_·7H_2_O, 1.3 μg/L Co(NH_3_)·H_2_O, 1.8 μg/L MnCl_2_·4H_2_O, 0.01 μg/L Vitamin B_1_, and 0.0005 μg/L Vitamin B_12_. Additional file 1: Table S1 shows the concentration of the main ions (> 1 mg/L) in the PEM medium. The pH value of the PEM medium was 3.6 adjusted with 3 M sodium hydroxide (NaOH) and 1 M hydrochloric acid (HCl). The microalgae cells were grown in 2-L glass column photobioreactors with a 10 cm internal column diameter. The photobioreactors contained 1.5 L PEM medium that was stirred with 0.2 μm-filtered mixed gas (2% CO_2_, v/v, the gas flow rate was 6 L/min) and illuminated with an LED lamp at a light intensity of 150 μmol photons m^−2^ s^−1^. The temperature of the cultivation remained 25 °C.

### The growth curve of *E. gracilis* and pH changes in culture conditions

To determine the flocculation method of pre-concentrated *E. gracilis* cells, each day, we measured the cells’ growth curve and pH changes in the PEM medium under the above culture conditions until the cells reached the plateau phase. The microalgae’s growth curve was monitored by measuring the absorbance at 750 nm (OD_750_) using a UV–Vis spectrometer (UV2350, UNICO, China) and the culture medium’s pH value was monitored with a pH meter (pH 30, Clean-Leau Instruments, China).

### pH-induced flocculation

As the pH of the medium increases and the flocculation efficiency (FE) of cells’ suspension reaches its maximum at a certain point in time (25 °C), the value of pH is a critical point, which is called CPV. To determine the CPV required for achieving a high FE (FE > 90%), 0.8 g/L biomass dry weight (DW) of microalgal suspensions was prepared in 45-mL centrifuge tubes. 3 M NaOH and 1 M HCl were used to adjust the pH value. The DW of microalgal cells was measured according to Lee et al. [42]. Experiments were conducted to determine the FE at pH values 3, 4, 5, 6, 7, 8, 9, 10, 11, and 12; at different time intervals of 10, 20, 30, 40, 50, and 60 min.

The FE of each suspension was calculated according to the following equation:

1 $$FE = \frac{{ODb - OD{\text{a}}}}{ODb} \times 100\%$$

OD_750_, the optical density by absorbance at 750 nm; where “*ODb*” is the OD_750_ of the cells’ suspension before pH adjustment and “*ODa*” is the OD_750_ after the complete treatment; all of “*ODb*” and “*ODa*” were measured from the center of the centrifuge tubes.

After verifying that the algae biomass FE was optimized at a basic pH, further study was conducted via precipitate and morphological cell analysis. A microscope (Leica DMi1, Leica Microsystems, Germany) was used to observe the morphological changes in cells from the different pH level experiments. Similarly, the FE of different algae biomass concentrations (Dry weight = 0.8, 1.3, 1.8, 2.3, and 2.8 g/L) was determined at 20, 40, and 60 min.

### Determination of the flocculation mechanism

Precipitates on the bottom of the centrifuge tubes and the turbidity of the solutions from the central of centrifuge tubes were observed and measured using a laboratory hazemeter (NDH2000, Shenzhen University, China) under the PEM-Mg^2+^ (without MgSO_4_·7H_2_O), PEM-Ca^2+^ (without CaCl_2_·2H_2_O), and PEM-Mg^2+^-Ca^2+^ (without MgSO_4_·7H_2_O, CaCl_2_·2H_2_O) after 1 h, respectively, to determine whether precipitates formed in the absence of Mg^2+^ or Ca^2+^, both Mg^2+^ and Ca^2+^, respectively, from the culture medium.

Next, to unequivocally demonstrate the role of divalent cations in the flocculation process, we assessed whether flocculation induced by the CPV could be inhibited by adding 30 mM EDTA, a chelating agent that can sequester polyvalent cations. This experiment was performed using four test groups: a control group A at the CPV without EDTA,a control group B with added EDTA; a solution at the CPV with added EDTA; and a solution at the CPV without added EDTA. After the algae cells under CPV conditions were removed by centrifugation and filtered using Whatman GF/C glass microfiber filters, the dissolved Ca^2+^ and Mg^2+^ concentrations from the center of the centrifuge tubes were analyzed. Meanwhile, the Saturation Indices (SI) and the logarithmic Solubility Product Constant (lgKsp) of compounds related to Ca^2+^ and Mg^2+^ in the PEM medium by pH 8.5, 25 °C treatment were predicted and analyzed with Visual MINTEQ, ver. 3.0 software.

To further investigate the relative importance of Ca^2+^ and Mg^2+^ in the CPV flocculation process, we tested whether flocculation at the CPV could be induced in a medium lacking Ca^2+^ or Mg^2+^. To do so, algae cells were separated from the medium using centrifugation and resuspended in a fresh medium without Ca^2+^ and Mg^2+^. In the first series, algae cells were resuspended in a medium without Mg^2+^ and containing varying Ca^2+^ concentrations of 0—(0 mM), 1—(0.2 mM), 2—(0.4 mM), and 4—(0.8 mM) fold that of the control group. In a second series, Ca^2+^ was omitted and Mg^2+^ was added in concentrations of 0—(0 mM), 1—(5.0 mM), 2—(10.0 mM), and 4—(20.0 mM) fold that of the control group. The FE of dissolved Ca^2+^ and Mg^2+^ concentrations of all the above groups were analyzed. Finally, the FE was determined under the PEM-PO_4_^3−^ [without KH_2_PO_4_, adding fourfold (0.8 mM) Ca^2+^ and (20.0 mM) Mg^2+^, respectively], PEM + PO_4_^3−^ (with 4.4 mM KH_2_PO_4_, adding fourfold Ca^2+^and Mg^2+^, respectively) and the control (without MgSO_4_·7H_2_O, CaCl_2_·2H_2_O) at the CPV. Dissolved Ca^2+^ and Mg^2+^ ion quantification was performed using inductively coupled plasma optical emission spectroscopy (ICP-OES) (Optima 7000 DV, PerkinElmer, USA). The specific steps were performed according to Trevelin et al. [43].

### Preliminary application of pilot-scale culture

The pilot-scale (60-L column photobioreactors) culture FE was determined by alkalescent pH 8.5 treatment at 0.5, 1, 1.5, 2, and 12 h. Next, the algae biomass DW was analyzed before and after flocculation, while chlorophyll production and paramylon production were analyzed at 2 and 12 h.

Chlorophyll production determination was conducted as described by Price [44], using the following modification. 1 mL cell suspension was mixed with 4 mL of acetone in centrifuge tubes that were cooled in ice water. After 1 h in the dark, the samples were remixed and centrifuged. The extinction of the clear suspension solution was determined with a UV–Vis spectrometer at 663 nm (the residue from a single extraction was colorless and further extractions did not increase the yield). The concentration and amounts of chlorophyll were then calculated from the extinction coefficient for chlorophyll, as reported by MacKinney [45].

Paramylon production quantification was described by Takenaka et al. [46] using the following modification. To improve the quantification accuracy, 30 mM of EDTA chelating agent was added to the cells’ suspension, in both the experimental group and the control group, to prevent the precipitation from affecting the paramylon content. After the cells’ suspensions were centrifuged and freeze–dried, ~ 5–10 mg of freeze–dried algae powder and 5 mL of acetone solution were transferred to a 15-mL centrifuge tube, and shaken for 10 s, then placed in a shaker for 2 h. After the tube was centrifuged at 2000*g* for 5 min, the supernatant was removed. 1.5 mL of 1% SDS solution was added to the tube, after which the contents were transferred into a 1.5-mL centrifuge tube and heated in a water bath at 85 °C for 2 h. Again, the supernatant was removed after the centrifuge tube was centrifuged at 2000*g* for 5 min. The precipitate was washed and centrifuged in 1 mL deionized water, then oven-dried at 70 °C to a constant weight. The obtained precipitate was paramylon. The paramylon production (g/L) was calculated as shown in Eq. (2):

2 $${\text{Paramylon production}} = \frac{PM}{AP}$$

where “*PM*” and “*AP*” are the dry weight of the paramylon and algae powder, respectively.

### Statistical analysis

Each growth curve, pH value, flocculation efficiency test, ion concentration, paramylon production, and chlorophyll content test were performed in triplicate and the average was reported. All data were statistically analyzed by Student’s *t* test analysis to investigate the difference compared to the control group. *p* values less than 0.01 (*p* < 0.01) were considered significantly different, *p *< 0.05 statistically different, and *p* values more than 0.05 (*p* > 0.05) not significant. Standard errors were included in all the graphs.

## Supplementary information

**Additional file 1: Fig. S1.** The flocculation efficiency of *E. gracilis* cells and the change in sedimentation under varying pH treatments. **Table S1.** Concentrations of the main ions (> 1 mg/L ) in the PEM culture medium.

## Data Availability

All data generated or analyzed in the present study are included in this article and in additional information.

## Abbreviations

EPA: Eicosapentaenoic acid
NaOH: Sodium hydroxide solution
HCl: Hydrochloric acid
CPV: The critical point of pH value
FE: Flocculation efficiency
PEM medium: Photoautotrophic euglena medium
ICP-OES: Inductively coupled plasma optical emission spectroscopy
LDWI: The Lifshitz–van der Waals interaction
EI: Electrostatic interaction
LABI: Lewis acid–base interaction
DOM: The dissolved organic matter
CCAP: Culture Collection of Algae and Protozoa
DW: Cellular dry weight
SI: Saturation index
IAP: Ion activity product
K_SP_: Solubility product constant
