# Supplementary material for: Pre-concentration of microalga Euglena gracilis by alkalescent pH treatment and flocculation mechanism of Ca3(PO4)2, Mg3(PO4)2, and derivatives
Source: Biotechnol Biofuels. 2020 May 29;13:98. doi: 10.1186/s13068-020-01734-8 (PMC7260821; doi:10.1186/s13068-020-01734-8)
Supplement: Supplementary file 1 — Additional file 1: Fig. S1. The flocculation efficiency of E. gracilis cells and the change in sedimentation under varying pH treatments. Table S1. Concentrations of the main ions (> 1 mg/L ) in the PEM culture medium. [file 13068_2020_1734_MOESM1_ESM.docx]

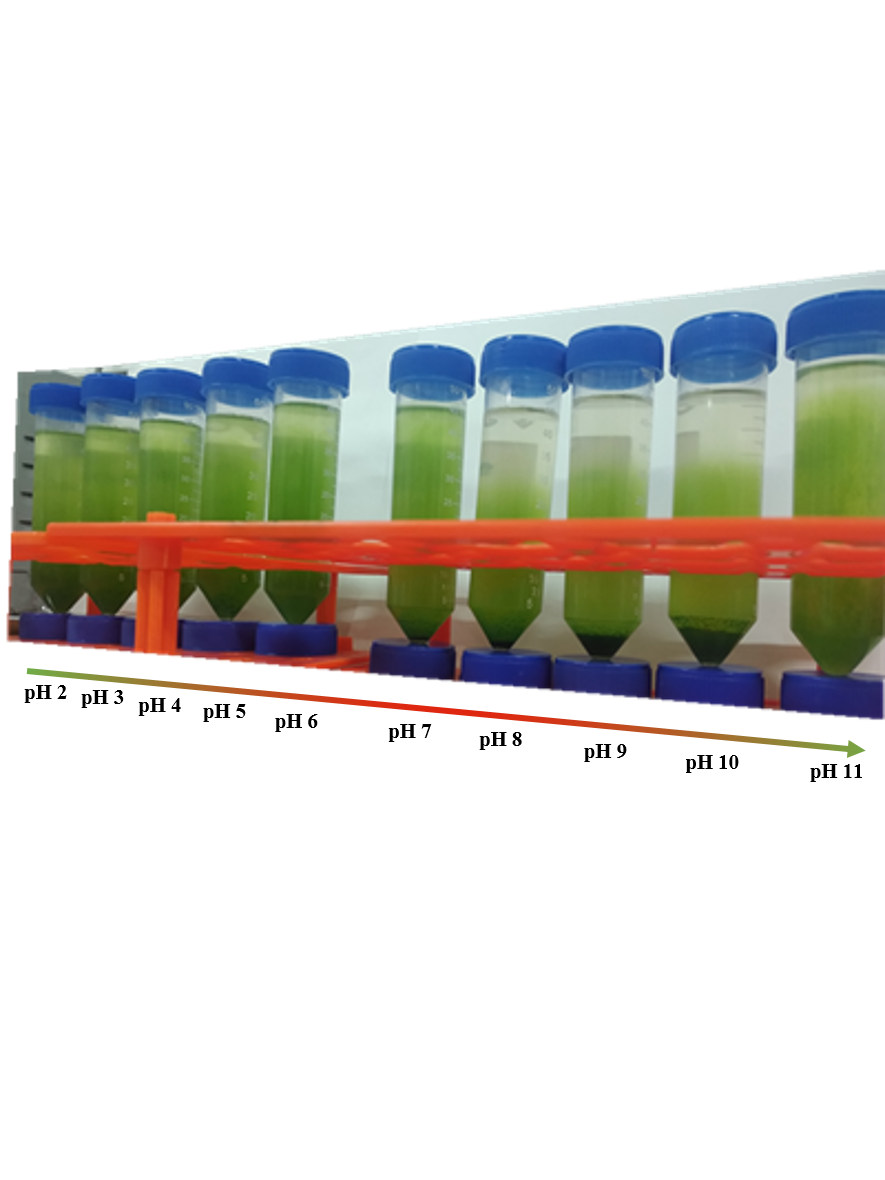


**Fig. S1** The flocculation efficiency of *E. gracilis* cells and the change in sedimentation under varying pH treatments.

**Table S1** Concentrations of the main ions ( > 1mg/L ) in the PEM culture medium.

| Ions | mg/L | mM |
| --- | --- | --- |
| Cl^-^ | 1199.64 | 33.84 |
| NH_4_^+^ | 605.72 | 33.65 |
| SO_4_^2-^ | 479.72 | 5.00 |
| PO_4_^3-^ | 418.84 | 4.41 |
| K^+^ | 171.95 | 4.41 |
| Mg^2+^ | 119.77 | 4.99 |
| Ca^2+^ | 7.21 | 0.18 |
